# Intravoxel Incoherent Motion Diffusion-weighted Magnetic Resonance Imaging for Monitoring the Early Response to ZD6474 from Nasopharyngeal Carcinoma in Nude Mouse

**DOI:** 10.1038/srep16389

**Published:** 2015-11-17

**Authors:** Yanfen Cui, Caiyuan Zhang, Xiaoming Li, Huanhuan Liu, Bing Yin, Tianyong Xu, Yong Zhang, Dengbin Wang

**Affiliations:** 1 Department of Radiology, Xinhua Hospital, Shanghai Jiao Tong University School of Medicine, Shanghai 200092, China; 2 MR Advanced Application and Research Center, GE Healthcare China, Shanghai 201203, China

## Abstract

Early therapeutic effects of anti-angiogenic agent ZD6474 upon nasopharyngeal carcinoma (NPC) in nude mouse were monitored by using intravoxel incoherent motion (IVIM) diffusion-weighted imaging (DWI). Mice bearing NPC underwent IVIM DWI at baseline and after 1, 3, and 7 days of treatment with ZD6474 or vehicle (n = 12 per group). Parameters of apparent diffusion coefficient (ADC), true diffusion coefficient (D), perfusion fraction (f), and blood pseudodiffusion coefficient (D*) at different time points were compared between the two groups or within the treated group. In the treated group, the perfusion-related parameters f and D* of the tumors decreased significantly on day 1 while the diffusion-related parameters ADC and D were significantly higher beginning on day 3 compared with the control group. The decreases in f on day 1 and D* on day 3 were moderately correlated with the smaller tumor size change on day 7. Moderate correlations were established between MVD and f and D* as well as between increased TUNEL or decreased Ki-67 index and ADC and D. This study supports that IVIM DWI is sensitive to detect the ZD6474-induced changes in NPC in nude mouse and the f parameter could predict early response to anti-angiogenic treatment.

Nasopharyngeal carcinoma (NPC) is one of the most common malignancies of the head and neck in Southeast Asia. Currently, the chemoradiotherapy is the treatment of choice for the NPC. However, radioresistance and tumor recurrence are major therapeutic hurdles in NPC^1^,^2^,^3^,^4^. Thus, some emerging therapeutic strategies, such as intensity-modulated radiation therapy (IMRT), immunotherapy, and anti-angiogenic therapy, have attracted more attention. Angiogenesis is the process leading to formation of novel blood vessels within tumors and plays an essential role in supporting tumor growth and the development of distant metastases. Therefore, targeting of angiogenic pathways could be and has been exploited as an effective strategy for cancer treatment^5^,^6^,^7^. Recently, Vandetanib(Zactima; ZD6474), an oral small-molecule multitargeted tyrosine kinase inhibitors (TKIs) that primarily targets vascular endothelial growth factor receptor (VEGFR) and epidermal growth factor receptor (EGFR) tyrosine kinase activity, has been revealed to exhibit anti-angiogenic and anti-proliferative effects in a panel of histologically diverse human cancer xenografts in nude mice, including NPC^8^,^9^,^10^,^11^,^12^.

Currently, the noninvasive imaging technologies for monitoring the early therapeutic effects for tumor have been widely implemented in the clinical study as well as in the experimental research. Among the imaging modalities and technologies, the magnetic resonance (MR) diffusion-weighted imaging (DWI) have been verified to effectively detect the *in vivo* water motion restriction by measuring the apparent diffusion coefficient (ADC), which can also reflect the tumoral response to treatment to some extent^9^,^13^,^14^,^15^,^16^. However, the ADC value obtained from DWI will intrinsically combine the effects of both diffusion and perfusion of the tissues. More recently, a promising functional imaging technique, intravoxel incoherent motion (IVIM) DWI, has been developed to permit the separate quantification of tissue perfusion and diffusion information. This Non-Gaussian models achieved significantly better fitting of DWI signal than the mono-exponential model and might reveal additional tissue properties beyond ADC^17^. By implementing a bi-exponential fitting model with multiple b values, perfusion-related parameters such as the perfusion fraction (f) and blood pseudodiffusion coefficient (D*), and diffusion-related parameters such as the true diffusion coefficient (D), can be separately measured^18^. Currently, IVIM DWI has been applied to the investigation of detection and characterization of renal, pancreatic, breast tumors, and so on^19^,^20^,^21^,^22^. In the realm of head and neck tumors including NPC, this technology has recently shown its potential value in evaluation of the imaging features, differential diagnosis, and the prediction of the therapeutic response of radiochemotherapy in clinical patients^17^,^23^,^24^. However, to the best of our knowledge, no studies have investigated the usefullness of IVIM to assess the therapeutic effects of anti-angiogenic agents upon the NPC in an experimental research^25^,^26^.

Therefore, the purpose of the present study was to investigate the feasibility of IVIM DWI to evaluate the early therapeutic effects of ZD6474 upon human NPC xenografts in nude mouse.

## Results

### Effects of ZD6474 on CNE-2 tumor growth

**Figure 1:**
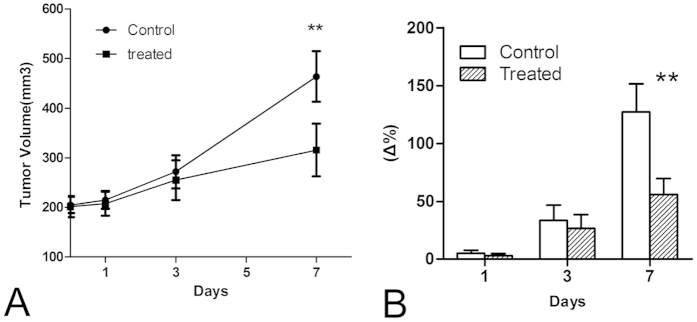
Tumor volume size (**A**) and percent changes (**B**) throughout the treatment week. **p < 0.01 versus the control. Error bars denote standard errors.

Oral administration of 100 mg/kg ZD6474 each day for 7 days was effective in delaying CNE-2 NPC tumor growth (Fig. 1). As shown in Fig. 1A, the time-dependent increase in tumor growth in the treated group was lower than that in the control group, and the tumor volumes in the treated group were significantly lower than those in the control group until day 7 (*P* < 0.01). Additionally, the average tumor growth rates, expressed as (V − V_0_)/V_0_ × 100%, were 5.0% ± 2.4% on day 1, 33.3% ± 13.2% on day 3, and 126.9% ± 24.5% on day 7 in the control group (Fig. 1B). In contrast, in the ZD6474-treated group, the relative changes in tumor volumes increased 2.8% ± 1.9%, 26.3% ± 9.1%, and 55.9% ± 13.7% on days 1, 3, and 7, respectively (Fig. 1B). The tumor volumes and growth rates in mice in the treated group were significantly reduced compared with those in the control group starting from day 7 (*P* < 0.01). No observable body weight loss and any other side effects were observed at the dosage of ZD6474 used in our study, indicating that this dosage was safe and nontoxic to mice.

### Imaging findings

**Figure 2:**
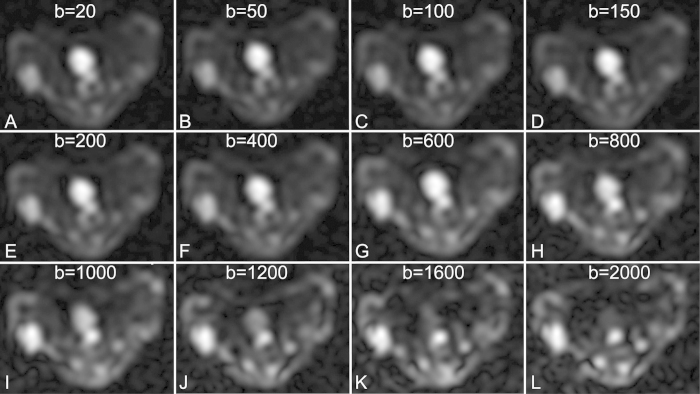
IVIM DW images with 12 b-values (**A–L**) for 0, 20, 50, 100, 150, 200, 400, 600, 800, 1200, 1600, and 2000 s/mm^2^.

**Figure 3:**
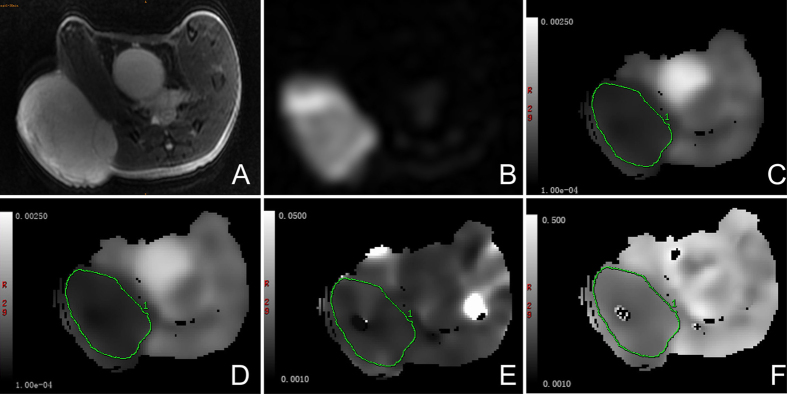
Axial T_2_-weighted image (**A**) and DW imaging with b value for 2000 s/mm^2^ (**B**) and the corresponding IVIM parameter maps (**C–F**) of one animal in the control group. (**C**) ADC map. (**D**) D map. (**E**) D* map. (**F**) f map. Regions of interest were manually circumscribed for the high-signal areas on lesions observed by DW imaging with a b value of 2000 s/mm^2^ for recording the corresponding IVIM parameter.

The source images of IVIM DWI with 12 individual b values from 0 to 2000 s/mm^2^ were successfully acquired for all experimental mice. As shown in Fig. 2, images with increasing b values show compromised tumoral anatomy and characterization, and it was possible to differentiate the viable tumors from necrotic tissues. Therefore, regions of interest (ROIs) were manually circumscribed for the high-signal areas on lesions observed under DWI with a high b value of 2000 s/mm^2^ for recording the corresponding IVIM parameters. As shown in Fig. 3, one tumor in the control group was clearly demarcated on T_2_-weighted MR images and DWI with a high b value, and the corresponding IVIM parameter maps were also obtained with moderate imaging quality.

### Reproducibility of ADC values and IVIM parameters

Analysis of D* and f values of six mice in the control group yielded moderate reproducibility, with CVs of 24.3% and 19.5%, respectively. The CVs of ADC and D were 7.2% and 5.1%, representing good reproducibility.

### Group analyses

**Figure 4:**
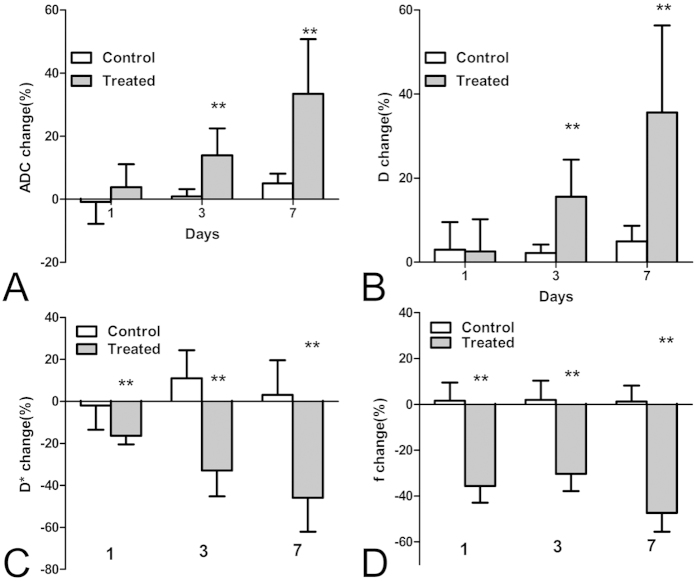
Bar graphs comparing the percent change in IVIM DWI parameters of the tumor, including (**A**) ADC, (**B**) D, (**C**) D*, and (**D**) f between treated and control groups at each time point after treatment. Comparisons between the two groups were performed using the Mann-Whitney test (*P < 0.05; **P < 0.01). Error bars denote standard errors of the mean.

The results of the absolute and percentage changes of ADC values and IVIM parameters of the NPC tumor for the control and treated groups at each time point are displayed in Table 1 and Fig. 4, respectively. For the control group, all the ADC values and IVIM parameters of the tumor remained relatively constant over a 7-day experiment, and the percentage changes in ADC and D values of the tumor in the control group gradually increased over time, reaching about 5% at day 7. However, in the treated group, both the mean value and relative changes in ADC and D values increased at each time point following daily treatment with ZD6474 and were both significantly higher than those in the control group starting from day 3 (P < 0.05; Table 1, Fig. 4A,B). For the perfusion-related parameters, the mean D* value in the treated group was lower than that in the control group at day 1, but did not reach statistical significance (*P* > 0.05; Table 1). In contrast, the relative change in D* value in the treated group decreased significantly compared with that in the control group within only 1 day (median, −16.69% versus −2.21%, *P* < 0.01; Fig. 4C). The mean and percentage of changes in f values of the tumor were significantly decreased in the treated group compared with those in the control group after only 1day (19.59 ± 4.08 versus 12.98 ± 4.37, respectively, *P* < 0.01; −37.77% versus −0.28%, respectively, *P* < 0.01; Table 1 and Fig. 4D).

### Serial measurements of all IVIM DW imaging parameters in the treated group

**Figure 5:**
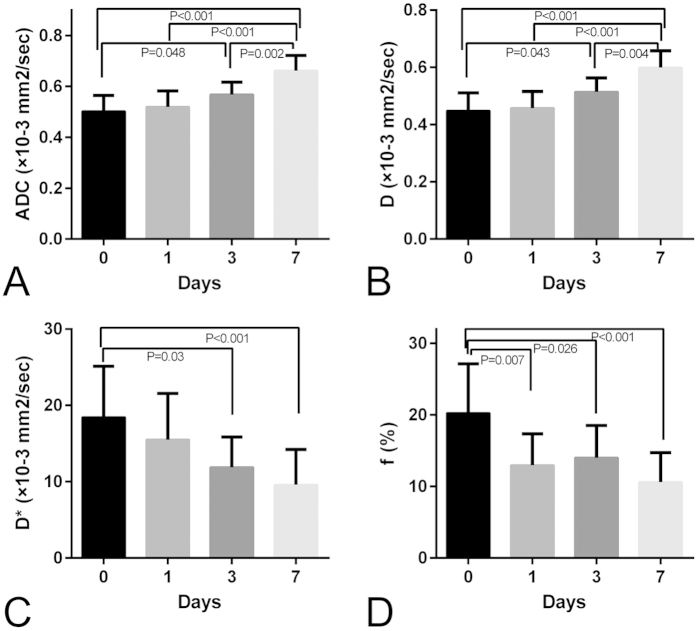
Box plots depicting the differences in the mean ADC and IVIM parameters of the tumor in the treated group at different time points. All data were analyzed using ANOVA, followed by testing with the Bonferroni test in cases of statistical significance.

To further explore the efficacy of ADC and IVIM parameters for monitoring the therapeutic effects of ZD6474 on NPC tumors in treated animals, serial measurements were carried out and parameter maps were constructed (Fig. 5). As shown in Fig. 5A,B, the ADC and D values in treated group increased slightly after 1 day of treatment, which significantly increased at days 3 (for ADC and D, *P* = 0.048 and *P* = 0.043, respectively) and 7 (both *P* < 0.001) compared with those at baseline. Serial measurements of the pseudodiffusion coefficient D* of the tumor in the treated group demonstrated that D* gradually decreased over the course of 7 days compared to that at baseline, but was not statistically significant after only 1day of treatment (Fig. 5C). In contrast, f decreased markedly at day 1, from a mean value of 20.24% to 12.98% (*P* < 0.001), followed by partial recovery on day 3, with a mean value of 15.17%, which was still lower than that at baseline (*P* = 0.026, Fig. 5D). On day 7, the f value decreased to a mean value of 10.63% again, which was significantly different from the baseline value (*P* < 0.001, Fig. 5D).

### Early prediction of tumor response based on the changes in ADC values and IVIM parameters

**Figure 6:**
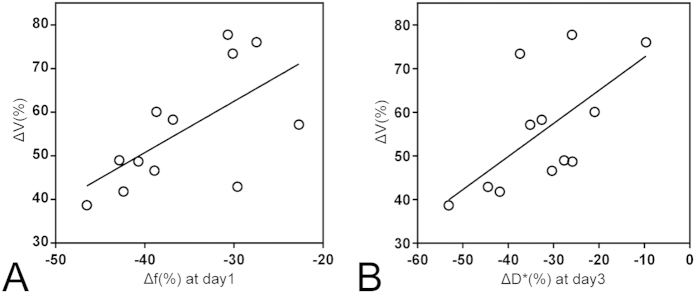
Graphs show correlations between changes in tumor size at the 7-day follow-up and relative changes in (**A**) f at day 1 and (**B**) D* at day 3 compared with baseline. Moderate correlations with r values of (**A**) 0.657 (*P* = 0.02) and (**B**) 0.58 (*P* = 0.048) were observed.

To determine the feasibility of IVIM DWI for predicting the early tumoral response to ZD6474, the correlation between the relative increases in tumor size at the end of the study and percentage changes in ADC and IVIM parameters of the tumors at 1 and 3 days of treatment were evaluated. Among these parameters, only percentage changes in f values at day 1 and D* values at day 3 were significantly correlated with tumor response, with a Spearman’s correlation coefficient r of 0.58 (*P* = 0.048, Fig. 6A) for f values and r of 0.657 (*P* = 0.02, Fig. 6B) for D* values. These data indicated that there was a substantial decrease in the f value at day 1 and a dramatic increase in the D* value at day 3, which could predict a smaller increase in tumor size at 7 days of treatment.

### Histological assessment of tumor response and its correlation with ADC and IVIM parameters

**Figure 7:**
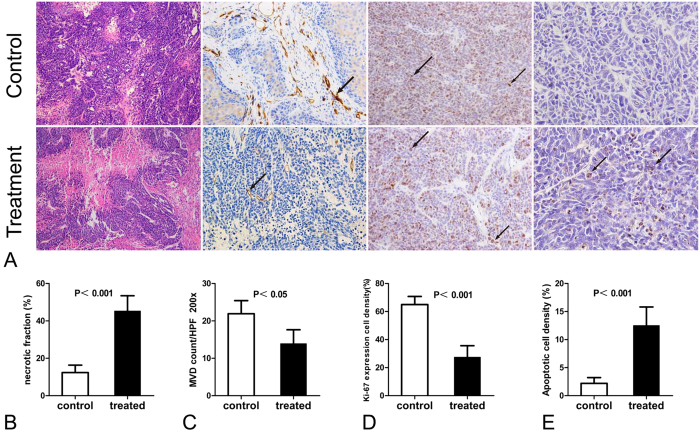
Histologic assessment of tumor response. (**A**) Representative HE-stained (original magnification, 200×), CD31-stained (200×), Ki67-stained (400×), and TUNEL-stained (400×) slices for the control and treated groups at the end of follow-up. Apoptotic cells and cells expressing Ki-67 are indicated with arrows. Bar graphs show the (**B**) necrotic fraction (NF), (**C**) microvascular density (MVD), (**D**) proliferating cell density (Ki-67 index), and (**E**) apoptotic cell density (TUNEL index) in untreated and ZD6474-treated tumors. Error bars denote standard errors of the mean.

Changes induced by ZD6474 treatment, as observed by IVIM DWI, were compared with the corresponding histology (Fig. 7 and Table 2). Hematoxylin-eosin stained sections from the control group showed a dense cellular pattern, while ZD6474-treated tumors showed a decrease in cellular density with more necrotic areas (Fig. 7A,B). Because of the anti-angiogenic effects of ZD6474, MVD scores in ZD6474-treated tumors were significantly decreased compared to those of tumors in the control group on day 7 (Fig. 7A,C). The density of cells showing Ki-67 expression in the treated group was significantly lower than that in the control group (Fig. 7A,D), while the density of apoptotic cells, as analyzed by using the TUNEL index, was significantly higher than that in the control group (Fig. 7A,E), indicating an effective therapeutic response.

The correlations between all IVIM DWI parameters and corresponding histological features, including MVD score and Ki-67 or TUNEL index were shown in Table 2. MVD was significantly correlated with the perfusion-related parameters f and D* (r = 0.47 [*P* = 0.02] and r = 0.67 [*P* < 0.001], respectively). However, no significant correlations were observed between MVD and the diffusion-related parameters ADC and D. The density of apoptotic cells was significantly correlated with ADC and D (r = 0.71 [*P* < 0.001] and r = 0.68 [*P* < 0.001], respectively). In contrast, Ki-67 expression of the tumor was inversely correlated with these diffusion-related parameters (r = −0.72 [*P* < 0.001] and r = −0.69[*P* < 0.001], respectively). Interestingly, Ki-67 expression was significantly correlated with the f value (r = 0.59, *P* = 0.002), and the TUNEL index was inversely correlated with the f value (r = −0.58, *P* = 0.003). However, no significant correlations were observed between D* and Ki-67 expression or TUNEL index (r = 0.40 [*P* = 0.051] and r = −0.40[*P* = 0.053], respectively).

## Discussion

The goal of the present study was to determine the feasibility of IVIM DWI for evaluation of the early therapeutic effects of ZD6474 upon the human NPC in nude mouse. Our study demonstrated that continuous administration of a biologically active dose of ZD6474 for 7 days significantly blocked tumor growth and that some IVIM parameters could predict serial pathophysiological changes in perfusion and diffusion of the tumors with moderate to good reproducibility.

Unlike conventional chemoradiotherapy, on one hand, the ZD6474 as an anti-angiogenic agents can cause cytostatic effects rather than cytotoxic effects, that is, anti-angiogenic treatment impede the tumor growth rather than directly kill the tumor tissue to reduce the tumor volume^27^, on the other hand, ZD6474 can inhibit VEGF-dependent tumor angiogenesis or vascular survival, resulting in a decrease in MVD and permeability, followed by ischemic changes and necrosis of the tumor^28^. Therefore, diffusion-related parameters ADC and D, which reflect the rate of microscopic water diffusion as a marker of cellular density or necrosis, would change later than perfusion-related parameters f and D*, which reflect the blood volume and velocity inside the tumors^18^,^29^. In our study, the perfusion-related parameters f and D* were significantly reduced in ZD6474-treated mice compared with those in the control group at the end of the study period, and these results corresponded to histopathological changes in MVD scores. Lower MVD scores in the treated group were considered to represent a downstream effect of angiogenesis, leading to lower f and D* values. Moreover, f and D* values were moderately correlated with MVD on day 7, and these results were similar to those obtained by Li *et al.*^30^, demonstrating that the perfusion-related IVIM parameters D* and f could be used for noninvasive evaluation of MVD inside the tumor. Therefore, among the IVIM DWI parameters, relative changes in f and D* values on day 1 could be used as early predictors of the anti-angiogenic response. In contrast, diffusion-related parameters, including D and ADC values, increased starting from day 3, which was later than the perfusion-related IVIM parameters. Furthermore, all of these changes in DWI parameters in response to anti-angiogenic treatment occurred before treatment-induced reductions in tumor size.

These results in our study were consistent with several previous dynamic contrast enhanced-MRI (DCE-MRI) findings, in which composite parameters, such as K^trans^ (the volume transfer constant) or Vp (the fractional plasma volume), which are influenced by vessel permeability, blood flow, and vessel surface area, were significantly reduced after administration of TKIs for several days or even weeks^9^,^27^,^31^,^32^. Although several previous study have demonstrated a high consistency among these parameters^33^,^34^,^35^, the exact links or differences between IVIM parameters and classical perfusion still remain unclear. Maïté *et al.*^25^ demonstrated that the f value is increased in patients with hepatocellular carcinoma (HCC) who responded to sorafenib treatment. This is consistent with decreased leakage and increased circulation, as suggested by the “normalization of tumor vasculature” theory, especifically, this suggests that improvement of the quality of the remaining tumor vessels results in decreased vascular permeability and interstitial fluid pressure. In our study, we did not find any evidence of morphological remodeling of the tumor vasculature except for the decreased MVD at the end of the study, which may contribute to the observed decease in the perfusion-related parameters f and D*. The relative increases in diffusion-related parameters, including ADC and D values, in the treated group compared with baseline may have been caused by ZD6474-induced intratumoral necrosis and apoptosis, as confirmed by the significant differences in histopathological results of the NF and TUNEL or Ki-67 indices between the control and treated groups. Moreover, the diffusion-related parameters ADC and D were moderately correlated with increased TUNEL index and decreased Ki-67 index, indicating the possibility of noninvasive evaluation of proliferation or apoptosis potential. These pro-apoptotic and anti-proliferative mechanisms may be attributable to a combination of direct inhibition of cancer cell growth by interfering with the EGFR autocrine pathway and the secondary anti-angiogenic effects of VEGFR-2 blockade in endothelial cells^8^.

Because TKIs continuously act on a variety of molecular pathways involved in tumor progression, imaging analysis should be performed within days instead of hours for the evaluation of changes in perfusion after ZD6474 treatment^26^. Our results revealed that the perfusion-related parameters f and D* were significantly reduced as early as the first day after initiation of ZD6474 treatment, though the MVD was not expected to be altered within one day in the acute treatment study. Thus, this observation may be attributed to some functional vascular effect, such as reduced blood velocity. The exact acute mechanism of ZD6474 within hours remains unclear but warrants further investigation. Our results also identified that percentage changes in f values on the first day of treatment and D* values on the third day of treatment were moderately correlated with relative changes in the final tumor volume on day 7 of treatment, indicating that f values have more potential to allow prediction of early favorable tumor response after ZD6474 treatment.

Our study had several limitations. First, the ADC values of the tumors were calculated using all 12 measured b values rather than the most commonly used two-point fit. In this way, the differences between the diffusion-related parameters ADC and D were decreased due to the reduction in the perfusion effect at low b values. Second, we just evaluated the correlation of IVIM DWI parameters with corresponding histologic features at the end of follow-up. Thus, we could not assess early histologic changes that might have explained the early changes in IVIM DWI parameters. Third, because of the heterogeneously distribution of tumor cell nest, hand-drawn ROI on one axial MR image of the tumor used in our study may bring about sampling bias and may be less representative than the three-dimensional measurements of the tumors.

In conclusion, the present study demonstrated that IVIM DWI is sensitive to detect the ZD6474-induced changes in human NPC in nude mouse, while the f parameter could allow for prediction of the early tumoral response to anti-angiogenic treatment.

## Methods

### Cell culture and animal models of NPC

All the experimental protocols were approved by the Ethics Committee of Xinhua Hospital Affiliated to Shanghai Jiao Tong University School of Medicine (Approval no., XHEC-F-2015-003) and were conducted in strict accordance with the Guidelines of the National Institutes of Health for the Care and Use of Laboratory Animals. CNE-2 cells (FDCC, Fu Dan IBS Cell Center), the poorly differentiated NPC cell lines, were cultured in RPMI 1640 supplemented with 10% fetal bovine serum (Gibco, Paisley, UK) and 1% penicillin/streptomycin at 37 °C in a humidified atmosphere with 5% CO_2_. Female BALB/c nude mice (n = 24, 6–8 weeks old, 18–22 g body weight) were obtained from Shanghai Experimental Animal Center (Shanghai, China) and were maintained in a specific pathogen-free environment. After a 1-week adaptation period, mice were subcutaneously injected with 2 × 10^6^ CNE-2 cells in 0.2 mL serum-free media into the right hind flank. Tumors were allowed to grow to 200 mm^3^ before treatment (approximately two weeks after implantation). To evaluate therapeutic response, the tumor growth curves for each experimental group were estimated by caliper measurement using the formula: V = 1/2*ab^2^ (a, length; b, width).

### Treatment

A total of 24 mice with CNE-2 human NPC tumors were randomly allocated to either the control group (n = 12) or the ZD6474-treated group (n = 12). ZD6474 (S1046, Selleckchem) was dissolved in a 1% (v/v) solution of polyoxyethylene sorbitan monoleate in deionized water. Mice in the treated group were administered with ZD6474 by oral gavage at a dose of 100 mg/kg once daily for 7 days, while the mice in the control group were administered with vehicle (sterilized water) at the same dose.

### MR imaging

For both the treated and control groups, MR imaging was performed immediately before treatment (day 0, baseline) and at 1, 3, and 7 days after treatment with ZD6474 or vehicle. All the experimental mice were anesthetized with an intraperitoneal injection of pentobarbital sodium (50 mg/kg body weight; Sinopharm, Shanghai, China). MR imaging (MRI) was performed for the NPC models on a clinical 3.0-T MRI system (SignaHDxt, GE Medical System, Milwaukee, WI, USA) with a custom-built-8-channel receiver coil with 2.5 cm inner diameter (Chenguang Medical Technologies Co., Shanghai, China). Transverse T_2_-weighted fast spin echo imaging (T_2_WI, TR/TE, 3000 ms/102.4 ms; matrix size, 64 × 64; field of view, 40 × 40 mm^2^; slice thickness, 1.2 mm; section gap, 0 mm) was performed. Subsequently, the IVIM DWI sequence was obtained using a single-shot echo-planar imaging with 12 b-values of 0, 20, 50, 100, 150, 200, 400, 600, 800, 1200, 1600, and 2000 s/mm^2^ (Fig. 1). The following parameters were used for this sequence: TR/TE, 3000 ms/102.4 ms; flip angle, 90°; matrix, 64 × 64; field of view, 80 × 80 mm^2^; section thickness, 2.4 mm; NEX, 4; and total scanning time, 6 mins and 35 s.

## Image analysis

### IVIM parametric map acquisition

Based on the bi-exponential fitting to IVIM model, the relationship between the signal variation and b value could be described by the following Equation[1]: S(I)/S(I_0_) = f exp(–b D*) + (1–f)exp(–bD), where S(I_0_) represents the signal intensity of the b value of 0, and S(I) signifies the measured signal intensity for a given b value^18^,^19^. D and D* are the true and pseudodiffusion coefficients, respectively, and f is the perfusion fraction linked to microcirculation. Because D* is significantly greater than D, its influence on signal decay can be neglected for b values greater than 200 s/mm^2^. Therefore, the D map was obtained by a least-squares linear fitting using high b values (400, 600, 800, 1200, 1600, and 2000 s/mm^2^), with the following simplistic Equation[2]: S(I)/S(I_0_) = exp (–b D). Then the D* and f values were calculated by substituting the D value into Eq. [1] by fitting all 12 b values corresponding signal intensities to Eq. [1]. The total ADC extracted from all 12 b values was measured by using mono-exponential fit: S(I)/S(I_0_) = exp(–b ADC).

All the IVIM images were transferred to a dedicated post-processing workstation (ADW4.3, GE Healthcare) for quantitative analysis. The mean values of all the IVIM parameters were measured independently by one radiologist (Zhang CY) with 5 years experience in MRI. After identification of the solid part of the tumor on conventional T_2_WI, regions of interest (ROIs) were manually drawn on the center slice for each tumor at each time point with DWI with a b value of 2000 s/mm^2^ to cover as much of the solid part of the tumor as possible and to avoid hemorrhagic, cystic, and necrotic areas. The IVIM parameter maps were generated automatically by the MADC program (Fig. 2), and the mean ADC, D, D*, and f values in the ROIs were obtained.

### Quantitative measurement

The change in tumor volume relative to the baseline was quantified for determining the treatment response as follows: Tumor growth rate = (Vol_given time_ − Vol_baseline_)/Vol_baseline_ × 100%, where Vol_given time_ was the tumor volume on day 1,3,5, or 7 and Vol_baseline_ was the tumor volume on day 0. For the ADC and IVIM parameters, percentage changes compared to baseline were calculated using the following equation: parameter value change = (Value_given time_ − Value_baseline_)/Value_baseline_ × 100%.

### Histological assessment

After the last MRI, all the experimental mice were sacrificed by cervical dislocation with deep anesthesia by means of intraperitoneal injection with pentobarbital sodium. Tumors were harvested, fixed in 4% paraformaldehyde overnight, dehydrated in 70% ethanol, and subsequently embedded in paraffin. Sections were then serially performed to approximately 4 μm thick with the same orientation as the MRI and were stained with hematoxylin and eosin according to the standardized procedures. The necrotic fraction (NF) of the tumor was defined as the percent area of necrosis relative to the total area of the tumor sections. Individual sections from each group were stained for apoptosis by using a terminal nucleotidyltransferase-mediated nick end labeling (TUNEL) assay (*In situ* Cell Death Detection Kit; Roche) and visualized with the peroxidase DAB method (DAKO, Denmark), followed by counterstaining with hematoxylin and eosin. Immunohistochemical staining for Ki-67 (1:200; Santa Cruz Biotechnology, CA, USA) and CD31 (1:200; BD Biosciences, CA, USA) was also performed. All quantitative analyses of the histological staining were carried out by using Image J software (http://rsb.info.nih.gov/ij). To obtain the histologic vascular parameters of the tumor, i.e., microvessel density (MVD), three hot spots (areas of higher vascular density compared with the rest of the tissue) were chosen at a low-power field (40×), and vessels were manually counted in a high-power field (200×) by a pathologist (Wang RF) with 10 years of experience in oncologic pathology. The MVD was calculated as the mean of three measurements in the hot spots.

### Statistical analysis

The reproducibility of ADC and IVIM measurements of the tumors was analyzed by repeating IVIM DWI at 6-h intervals for six randomly selected experimental mice from the control group and calculating the coefficients of variation (CVs). According to previous studies, CVs of below or equal to 10%, 11%–24%, and greater than or equal to 25% represent good, moderate, and poor reproducibility, respectively^26^,^32^.

All numerical values were expressed as means ± standard deviations. For comparisons between the control and treated groups, the Student’s t test was used for analysis of the mean values of ADC and IVIM parameters and all histopathological indices of the NPC tumors in nude mice, while the Mann-Whitney test was used to analyze the relative changes in ADC and IVIM values. For the treated group, serial relative changes in ADC and IVIM-derived parameters of the tumors at each time point were evaluated by using one-way analysis of variance (ANOVA) followed by the Bonferroni test for multiple comparisons. Spearman’s rank correlation test was performed for correlations between the percent changes in IVIM DW parameters and the percent changes in tumor size at the end of follow-up and for correlations between histological features, including MVD and Ki-67 or TUNEL indices and corresponding IVIM parameters. Differences with *P* values of less than 0.05 were considered statistically significant. All statistical analyses were performed with SPSS version 19.0 for Microsoft Windows (SPSS, Inc., Chicago, IL, USA).

## Additional Information

**How to cite this article**: Cui, Y. *et al.* Intravoxel Incoherent Motion Diffusion-weighted Magnetic Resonance Imaging for Monitoring the Early Response to ZD6474 from Nasopharyngeal Carcinoma in Nude Mouse. *Sci. Rep.* **5**, 16389; doi: 10.1038/srep16389 (2015).

## Figures and Tables

**Table 1:** Comparison of IVIM DW imaging parameters between the treated and control groups.

	**Day 0**	**Day 1**	**Day 3**	**Day 7**
ADC (×10−3 mm^2^/sec)				
Control	0.505 ± 0.053	0.500 ± 0.054	0.509 ± 0.058	0.530 ± 0.055
Treated	0.502 ± 0.062	0.520 ± 0.063	0.568 ± 0.049*	0.662 ± 0.060**
D (×10−3 mm^2^/sec)				
Control	0.442 ± 0.059	0.454 ± 0.049	0.452 ± 0.058	0.464 ± 0.060
Treated	0.448 ± 0.063	0.458 ± 0.058	0.514 ± 0.049*	0.599 ± 0.059**
D*(×10−3 mm2/sec)				
Control	16.79 ± 8.89	17.15 ± 10.53	18.02 ± 8.85	16.81 ± 8.88
Treated	18.44 ± 6.67	15.52 ± 6.04	11.87 ± 3.98*	9.59 ± 4.62**
f (%)				
Control	19.54 ± 5.02	19.59 ± 4.08	19.60 ± 3.86	19.58 ± 4.27
Treated	20.24 ± 6.90	12.98 ± 4.37**	14.00 ± 4.51**	10.63 ± 4.10**

Note: data was expressed by mean ± standard deviation. Comparisons between control and treated groups at the same time were performed by using the Student’s t test (*P < 0.05; **p < 0.01).

**Table 2:** Correlation between Histologic Features and ADC and IVIM Parameters at the 7-Day Follow-up (n = 24).

**Parameters**	**MVD**		**Ki-67**		**TUNEL**	
	**Spearman r**	**P value**	**Spearman r**	**P value**	**Spearman r**	**P value**
ADC	−39.6	0.055	−72.0	<0.001	71.4	<0.001
D	−36.4	0.080	−68.8	<0.001	67.9	<0.001
f	47.2	0.020	59.2	0.002	−58.0	0.003
D*	66.6	<0.001	40.4	0.051	−40.0	0.053

Note: All P values were calculated with the Spearman rank correlation test.
